# Immune Cell Therapy Promises More Effective Cure for Medulloblastoma

**DOI:** 10.3390/jpm16060326

**Published:** 2026-06-18

**Authors:** Marco Agostini, Pietro Traldi, Mahmoud Hamdan

**Affiliations:** Corso Stati Uniti 4, Istituto di Ricerca Pediatrica Città della Speranza, 35100 Padova, Italy; m.agostini@unipd.it (M.A.); mhglaxo@gmail.com (M.H.)

**Keywords:** medulloblastoma, immune checkpoints, MB resistance, mass spectrometry-based proteomics, CAR T and CAR NK cell therapy

## Abstract

Medulloblastoma is one of the most prevalent pediatric brain tumors. Currently, existing therapies for this devastating type of cancer can only prolong survival time with severe side-effects and relapse. These therapies are not curative for almost a third of treated patients, while most survivors are condemned to a poor quality of life. The addition of immune checkpoint inhibitors (ICIs) to immune therapy has given some hope to those suffering from this type of cancer. Although ICIs provide a valuable contribution to immunotherapy, the exploitation of immune checkpoint inhibition within existing therapeutic strategies to cure Medulloblastoma remains understudied. However, the identification of the main molecular subgroups of medulloblastoma is considered one of the success stories of oncology. This advancement in molecular profiling of MB paved the way to subgroup-directed clinical trials, which may lead to efficacious immune-targeted therapy. However, this relatively new development is still hampered by a substantial biological heterogeneity of the disease and the absence of a full understanding of the various mechanisms behind its resistance to existing therapeutic modalities. The inclusion of chimeric antigen receptor (CAR) T and CAR NK cell therapy within various therapeutic strategies and ongoing clinical trials has given fresh hope those suffering from this fatal disease. However, ongoing clinical trials suggest that this highly promising therapy can be impaired by a number of serious limitations, including cytokine release syndrome, Graft-versus-host disease, the scarcity of target antigens, and severe adverse events. Some of the ongoing clinical trials also suggest that CAR NK is less prone to some of these limitations. This review also highlights the contribution of mass spectrometry-based proteomics, and the increasing role of liquid biopsy rather than tissue biopsy.

## 1. Introduction

Medulloblastoma (MB) is one of the most prevalent pediatric brain tumors, which makes up about 20% of all brain cancers in children. This fatal disease affects six in one million children worldwide [1]. Current multimodal therapy combines surgery, craniospinal irradiation, and various chemotherapeutic agents. This therapeutic modality can cure about 70% of the treated patients [2,3]. However, the same therapy leaves the survivors with severe consequences for their health and their lifestyle. That said, we have to bear in mind that the wide variation in the three components of the therapy impacts on the severity of the consequences of the therapy. For example, the dimensions of the irradiated area, doses of chemotherapy, and the number of cycles will depend on the individual patient and on the subgroup to be treated.

Over the last ten years, there have been a number of developments, which may contribute to current research efforts to develop new and more efficacious immune therapy for pediatric MB. The first development is an accurate classification of the molecular subgroups of the disease. Such classification is fundamental for a reliable stratification of MB patients, more accurate prognosis of the disease, monitoring of the patient’s response to therapy, and a better understanding of the various mechanisms of resistance to immune therapy [4,5,6]. The current literature reports four medulloblastoma molecular subgroups: WNT, SHH, group 3, and group 4, with each group being defined by its characteristic genome-wide transcriptomic [7,8] and DNA methylation profiles [9,10]. In a recent study it was reported that the highest incidence rate of metastatic disease was associated with group 3 (~50% of cases) and group 4 (~30% of cases), WNT: ~10%, while the metastatic rates associated with the SSH group were variable, with worse outcomes observed with *TP53* mutations [11]. The final count of the subtypes associated with each subgroup is still under investigation. More recent studies using transcriptome profiling and large-scale methylation identified four subtypes of SHH and eight subtypes of non-WNT/non-SHH MBs [12,13].

The second and equally important development is the increasing use of liquid biopsies [14] in clinical sittings, with a clear shift from tissue biopsies to liquid biopsies sampling. The use of the latter method facilitates noninvasive and repetitive (longitudinal) sampling throughout the course of the disease. These characteristics become more attractive when we are dealing with MB patients with an average age of below nine years. Tissue biopsy has been, and remains, the gold standard for the diagnosis and molecular profiling of most types of cancer. This sampling method enjoys a high level of laboratory standardization, and it can furnish reproducible and accurate results. However, the same method has a number of limitations, including invasiveness, and the method cannot be performed in all anatomical sites, particularly in the case of brain tumors. These limitations can be mitigated, or even resolved, through the use of liquid biopsy sampling. This emerging sampling method has a number of advantages: noninvasive, no limitation on the sampling frequency, it facilitates continuous monitoring of the disease, and the acquired samples contain molecules/cells associated with genetic, epigenetic, and proteomic changes provoked by the tumor. These molecules include circulating tumor cells (CTCs), circulating tumor DNA (ctDNA), RNA, extracellular vesicles, proteins, and metabolites. Until recently, the low concentration within the tested biofluids represented an obstacle for obtaining meaningful information. The unprecedented advances in genomic and proteomic technologies facilitated high sensitivity and high resolution to allow the detection of extremely low levels of various analytes within various biofluids. These technologies include next-generation sequencing (NGS), also known as massively parallel sequencing [15], droplet polymerase chain reaction [16], beads emulsification amplification, and magnetics PCR (BEAMing PCR) [17]. Given the pathological characteristics of medulloblastoma and its site close to the spinal cord, liquid biopsy sampling of cerebrospinal fluid (CSF) and subsequent analysis can furnish valuable information on the evolution of the disease. MB cells are known to be disseminated through this fluid to distant locations in the brain and in the spinal cord. The use of mass spectrometry-based proteomics to characterize protein profiles within CSF and other biofluids derived from both patients and controls can provide much-needed information on biomarkers of the disease. A number of examples on the emerging role of liquid biopsy in the investigation of MB are given in the discussion.

The third and equally important development is the emerging role of CAR T-cell therapy. In this innovative method, cytolytic T cells are armed with a receptor that can recognize a surface protein on tumor cells. However, the successful application of this innovative method in solid tumors treatment is hampered by the low-level expression of surface antigens by this class of tumor. High density expression of such proteins is necessary for an optimal CAR activation. This observation may explain the success of this method in treating relapsed pediatric acute lymphoblastic leukemia, while such success has not been repeated with solid tumors. It can be said that targeting immune checkpoints as part of some therapeutic strategies to treat MB is still in its infancy. There is at least a dozen of identified human checkpoints (see Box 1), yet existing clinical trials have focused on a very limited number of the listed checkpoints. B7-H3 is one of few immune checkpoints, which is expressed with sufficient density in pediatric medulloblastoma [18]. Such high expression was found in about 96% of MB pediatric patients, and the same study showed that such expression was more frequent with group 4 patients compared with other subgroups. Although this study was conducted on a fairly small number of patients, the high levels of B7-H3 reported in this study confirmed the identification of this molecule by earlier studies as a promising target for CAR T-cell immunotherapy [19]. The last five years have witnessed a clear shift from CAR T to CAR NK cell therapy, in particular in solid tumors. More details on CAR T, NK are given in later sections.

Box 1 **Immune checkpoints undergoing various investigations and different clinical trials. Immune checkpoints 1–4 are the most researched, and were the first to be targeted with ICIs, while the others (5–11) are described as emerging checkpoints, some of which are undergoing various clinical trials, in which they are targeted with bispecific antibodies, drug conjugate antibodies, and more recently CAR T and CAR NK cell therapy.**

1. Cell death ligand-L1(PD-L1)

2. Programmed cell death-1 (PD-1)

3. Cytotoxic T lymphocyte antigen-4 (CTLA-4)

4. Lymphocyte activation gene-3 (LAG-3)

5. B7 homolog 3 protein (B7-H3)

6. B and T lymphocyte attenuator (BTLA)

7. V-domain immunoglobulin suppressor of T cell activation (VISTA)

8. T cell immunoglobulin and ITIM domain (TIGIT)

9. T cell immunoglobulin and mucin-domain containing-3 (TIM-3)

10. Inducible T cell costimulatory (ICOS)

11. CD155

### 1.1. Mass Spectrometry-Based Analysis

Mass spectrometry (MS) analysis of a targeted protein within a biological sample can yield accurate information on its level of expression, its post-translational modifications (PTMs), and its interaction and/or complexation with other proteins within the investigated sample. Strong evidence in the current literature indicates that level of expression, as well as certain PTMs, are two parameters which directly impact on the role of certain immune checkpoints in various tumors, including pediatric solid tumors [20,21,22]. Despite the well-demonstrated capabilities of MS-based proteomics, clinical applications of this technology remain very fragmentary. That said, the last few years have witnessed an increased application of this powerful technique in an increasing number of clinical trials searching for disease biomarkers and new therapeutic targets. This enhanced use of MS in clinical investigations can be attributed to a number of relatively recent developments: availability of high resolution, high mass accuracy instruments, and soft and more efficient ion fragmentation methods, which contribute to a more efficient detection of PTMs and their sites, and the introduction of more powerful software packages, allowing easier and more efficient data analysis, as well as enhanced contents of protein data bases, allowing the more accurate assignment of the investigated proteins. Real world examples of the role of mass spectrometry in analysis relevant to pediatric MB are discussed in more detail in a later section. The main steps in the MS-based platform and the application of this technique in clinical applications have been given in a number of recent articles [23,24,25,26].

Over the last ten years, CAR T-cell therapy has demonstrated encouraging efficacies in the fight against certain types of cancer, and, so far, seven CAR T-cell products have been approved by the U.S. Food and Drug Administration (FDA), and six by the European Medicines Agency (EMA) for the treatment of hematologic malignancies [27]. CARs are engineered receptors made up of two main components: the ectodomain, which is on the outside of the cell. This domain is a ligand-specific extracellular domain consisting of a single-chain variable-fragment (scFv) region and a hinge [28,29]. The scFv is a fusion protein of the variable regions of the light and heavy chains of immunoglobulins linked by a short flexible peptide linker [30]. The second component is the endodomain, which lies inside the cell and has the role of relaying various signals from the outside to the inside of the cell. This domain may consist of the intracellular T cell activation domain of CD3ζ as a single entity or by one or more intracellular co-stimulatory (or activation) domains [31]. In the present-day literature, CAR-T cells are classified into five generations based on the endodomain [30,32,33].

The impressive efficacy of Autologous CAR T-cell therapy in managing hematologic malignancies in clinical settings has been overshadowed by its high costs, excessive production times, and stringent patient selection. These limitations have resulted in an enhanced experimentation of allogeneic CAR T therapy [34,35]. This therapy, however, faces two major immunologic limitations: the risk of graft-versus-host disease induction by the allogeneic cells that recognize host tissues, and the rejection of the CAR-modified cells by the host immune system. In more recent years, CAR NK cell therapy has emerged as a possible alternative to CAR T therapy. Early clinical trials suggest that the use of NK cells may offer a number of advantages in terms of safety and versatility. That said, it is too early to make a rational comparison between the performance of CAR NK and CAR T therapies. The current literature reports over 1000 clinical trials using CAR T-cells therapy, while the number of clinical trials [36] using CAR NK is around 100.

### 1.2. Mass Spectrometry/Liquid Biopsies Investigation of Medulloblastoma

Low mutation rates in malignant pediatric medulloblastoma (MB) are one of the reasons for the lack of therapeutic targets for MB therapy. Advances in mass spectrometry-based proteomics have provided another research route for the identification of new therapeutic targets. As well as the search for therapeutic targets, the same technology is strongly involved in the search for all forms of biomarker for the prediction, diagnosis, and prognosis of various diseases, including MB. These biomarkers can give much-needed information on residual disease, response to therapy, patients’ stratification, and recurrence. Cerebrospinal fluid (CSF) is a rich source of biomarkers for brain tumors. To appreciate the type of information, which can be gained through the MS-based analysis of CSF, a number of real-world examples are discussed below.

Liquid chromatography coupled to tandem mass spectrometry (LC-MS/MS) was used to investigate protein profiles within CSF biopsy samples derived from medulloblastoma patients [37]. Samples were obtained from 34 MB patients and 19 healthy controls. The authors reported the quantification of over 1000 proteins in each CSF sample, a few of which were further validated using enzyme-linked immunosorbent assay (ELISA). These validated proteins included TKT, found to be highly upregulated in MB samples. Transketolase (TKT) is a key enzyme of the oxidative pentose phosphate pathway (PPP), encoded by the *KTK* gene. This enzyme is known to connect this pathway to glycosis, and its overexpression has been associated with tumorigenesis through diverse mechanisms [38,39]. The role of TKT in acute lymphoblastic leukemia, a serious pediatric blood cancer, was confirmed in another study [40]. The authors reported that regulating TKT activity inhibits the proliferation of human acute lymphoblastic leukemia cells.

In another study, LC-MS/MS was used to investigate the proteome within CSF waste from extra-ventricular drainage [41]. In their study, the authors acquired samples from 29 children suffering from various forms of brain tumor, and 17 healthy controls. The authors identified a similar number of proteins, approximately 1500 in both controls and in brain tumor patients. The main objective of this study was to identify protein biomarkers to discriminate patients from controls. Proteins identified by LC-MS/MS were further validated using the ELISA assay. These proteins included S100 protein B, Thymosin beta-4, and CD109. The results generated in both studies can be considered a useful contribution to assess the proteomic profile within CSF samples from both patients and controls. However, such an assessment has to take into consideration the following observations: First, protein concentration within CSF is known to be much lower than its counterparts in plasma, and such difference is attributed to the failure of many proteins to cross the blood–brain barrier [42]. Given the relatively simpler plasma sampling and its higher protein content compared to CSF sampling, the parallel use of both methods is likely to provide a richer, more informative proteome profiling of the investigated samples. Second, it is commonly assumed that certain proteins within CSF samples are shed into this biofluid by tumoral cells. Such an assumption cannot be fully verified unless tissue biopsies are examined. Such an option is not easy to perform, particularly at the average age of medulloblastoma pediatric patients.

Mass spectrometry-based proteomics was used to investigate molecular heterogeneity within medulloblastoma subgroups [43]. The authors examined the global proteome and phosphor proteome in a number of medulloblastoma samples. These analyses showed that tumors with similar RNA expression vary extensively at the post-transcriptional and post-translational levels. The same investigation revealed that post-translational modifications associated with *MYC* are indicative of a poor outcome in group 3 MB patients.

The above MS-based investigations raise an obvious question: in what way do such data contribute to research efforts to better understand MB? The answer to this question has to go beyond the simple identification of a high number of proteins within a single CSF sample. For example, the genes, which express what the authors called ‘potential biomarkers’ should be identified and analyzed to establish any mutations. In other words, to enhance the value of the generated proteomic profiles they have to be considered with the relevant genetic, epigenetic, and transcriptomic data. Such an inclusive approach of data generated by different technologies is imperative for a better understanding of MB. It is hoped that such an understanding may lead to deciphering the mechanism(s) of resistance of MB, which is considered one of the main challenges for the discovery of an effective curative therapy.

### 1.3. Mechanisms of Resistance in Medulloblastoma?

Currently, both histological and molecular heterogeneity in MB is considered the main challenge to present efforts to identify the likely mechanism(s) behind the resistance of MB to therapy. The World Health Organization (WHO) recognizes four molecular subgroups (mentioned earlier) and three histological subgroups: large cell anaplastic, desmoplastic/nodular, and medulloblastomas with extensive nodularity [44,45]. Among the four major molecular subgroups, group3 tumors exhibit high levels of *MYC* oncogene and high rates of metastasis. The current standard care of this subgroup consists of surgical resection, radiation, and multi-agent chemotherapy. Despite such aggressive treatment, patients of this subgroup tend to develop more aggressive recurrent forms of the disease with poor survival [46]. It is disappointing to note that resistance to these aggressive forms of therapy is also encountered in two emerging forms of immune therapy, CAR T [47,48] and immune checkpoint inhibitors [49], yet both forms of therapy have been demonstrated to be highly effective in treating some serious forms of cancer (for example, acute lymphoblastic leukemia).

**Figure 1:**
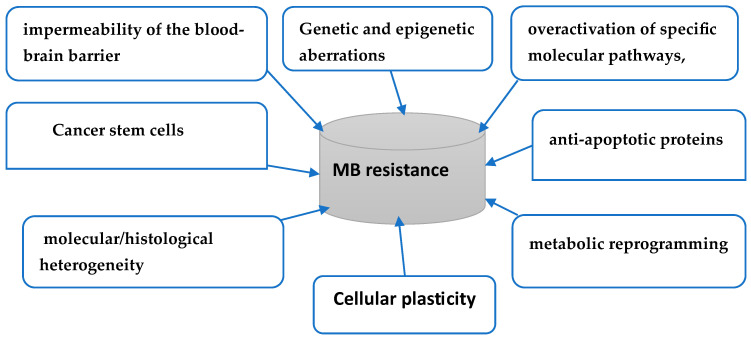
Some potential drivers behind medulloblastoma’s resistance to therapy.

The multifactorial nature of MB resistance is clearly evident in Figure 1. The current understanding of these drivers remains partial and needs to be expanded to allow an informed search for an effective cure for this devastating disease. Table 1 gives some brief observations and useful references on the drivers in Figure 1. Our knowledge of the individual and/or collective role of each of these drivers in various mechanisms of resistance remains unclear. However, various studies, together with numerous clinical trials, have underlined the relevant role that BBB plays in medulloblastoma resistance to therapy. BBB, specifically blood–brain–tumor barrier (BBTB) is a highly heterogenous barrier, and its permeability can vary, depending on the subgroup of MB [50]. Various studies have demonstrated that, among the major subgroups of MB, the WNT subgroup displayed a more favorable response to therapy [51,52]. These observations were confirmed using contrast-enhanced magnetic resonance imaging (CE-MRI) using a gadolinium-based contrast agent [53]. To evaluate the treatment of WNT-medulloblastoma, two patient cohorts from two children’s hospitals were investigated. The first cohort included 43 patients, while the second included 45 patients [54]. This study concluded that the medulloblastoma genotype dictates the BBB phenotype. The same study also demonstrated that the BBB was intact in the SHH medulloblastoma, underlying the observations that a wide range of therapeutic drugs are likely to be ineffective against this subgroup. These conclusions were based on the following results: 90% of primary WNT-medulloblastomas were hemorrhagic at surgery compared with subgroups SHH (12.5%), 3 (9%), and 4 (0%).

### 1.4. Different Response of Medulloblastoma Subgroups to Therapy

Large scale genomic sequencing identified four major molecular subgroups of medulloblastoma, namely Wingless (WNT), Sonic Hedgehog (SHH), Group 3 (Gp3), and Group 4 (Gp4). These subgroups have been further subdivided into a total of 13 subtypes with distinct molecular and clinical features [69,70]. Various preclinical studies and clinical trials have demonstrated that these subgroups respond differently to the various modalities of cell therapy. For instance, G3 is known to be the most aggressive, often showing poor responses due to a highly immuno-suppressive tumor microenvironment. There is strong evidence that the WNT subgroup has the most favorable prognosis among MB subgroups, and this subgroup is known to comprise about 10% of all MB cases [71]. The favorable prognosis of this subgroup is mainly attributed to the mutant β-catenin-driven vascular window, which disrupts the blood–brain barrier and significantly enhances the penetration of various therapeutic agents [54]. Subgroups 3 and 4 are both known to present with metastases at the time of diagnoses [72]. The first subgroup accounts for approximately 25% of cases, predominantly affects infants, and is characterized by *MYC* amplification [73], resulting in the poorest prognosis. Subgroup 4 constitutes about 35% of cases, is more prevalent in older children, and is associated with the presence of isochromosome 17q (i17q) in 80% of instances, leading to an intermediate prognosis with a five-year overall survival rate of 70% [74].

Current therapeutic modalities are prepared according to molecular classification. For example, infants with Group 3 tumors receive high-dose chemotherapy accompanied by autologous stem cell rescue to postpone radiotherapy [75]. Numerous clinical trials have confirmed that subgroup3 is the most aggressive, and nearly all patients present with metastases upon recurrence. Existing therapies for this group have not been demonstrated to confer a survival benefit at the time of recurrence. That said, there are various research efforts to identify biomarkers, which may refine patient stratification and a better selection of treatment. An example of such efforts is given in a recent study [75]. The authors compared recurrent tumors with newly diagnosed tumors and found that recurrent tumors had an elevated expression level of carbonic anhydrase 4. The same authors suggested that the genetic and pharmacologic modulation of carbonic anhydrase 4 could promote or reduce resistance to radiotherapy. Subgroup 4 is characterized by a low rate of actionable somatic mutations, which limit patients of this group to the existing aggressive regime, which includes surgery, radiation, and chemotherapy. Risk-adopted regimens did not give an improved outcome [76]. Subgroup 4 medulloblastoma is the most common and least biologically understood molecular subtype, accounting for about 35–40% of cases. Because these tumors have a low rate of actionable somatic mutations, current therapies still rely on an existing therapeutic regimen (surgery, radiations, chemotherapy).

### 1.5. Emerging Therapeutic Strategies for Treating Medulloblastoma

Currently, medulloblastoma is treated with aggressive therapeutic modalities, including surgery, chemotherapy, and craniospinal irradiation. In general, patients cured by these modalities tend to suffer lifelong severe side effects. It has been evident for a number of years that these modalities have to be replaced with more curative and less aggressive therapies. These promising therapies include immune cell inhibitors (ICIs), antibody–drug conjugates [77,78], CAR T-cells, CAR-natural killer (NK) cells, and immune cell engagers [79].

#### 1.5.1. Targeting Immune Cell Checkpoints in Medulloblastoma

**Figure 2:**
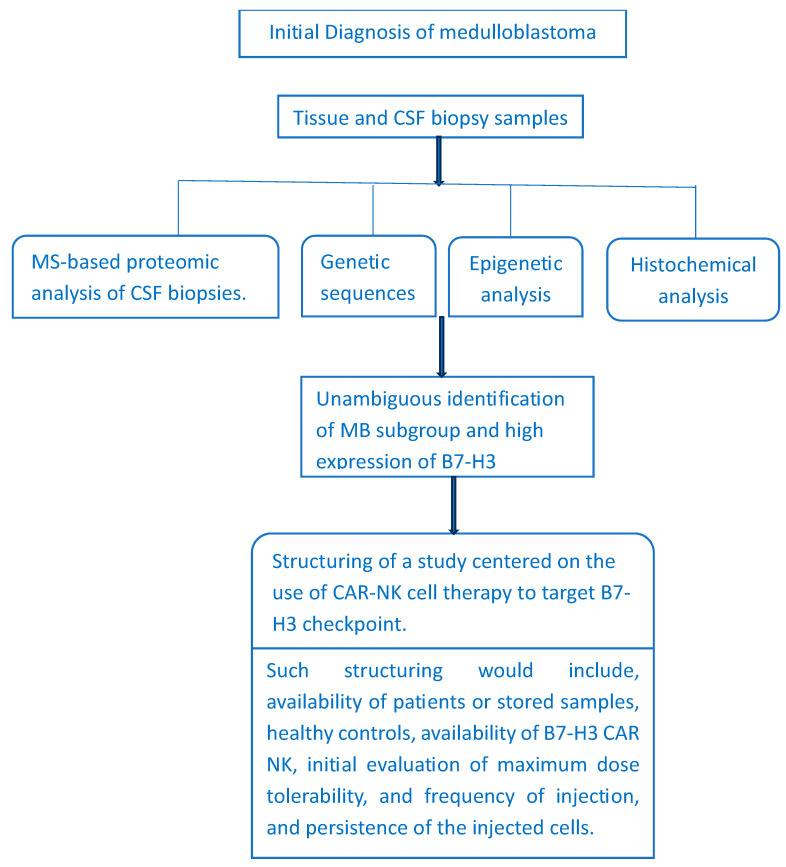
Schematic representation of the various steps leading to decision-making leading to the design of a clinical trial based on cell therapy. CSF analyses provide the proteomic profile and other biomolecules shed by the disease into the CSF. Genetic sequences provide targetable mutations, while epigenetic analyses should identify relevant dysregulations associated with the disease. The level of expression of B7-H3 is measured by histochemical staining of the investigated tissues.

The high expression of most of the checkpoints listed in Box 1 has been linked with the initiation and/or progression of various forms of cancer. The case of medulloblastoma is rather interesting, where the only checkpoint expressed with sufficient intensity is B7-H3. Furthermore, the same protein is known to be expressed on both medulloblastoma cells and on non-tumor cell types within the tumor microenvironment (TME). Targeting B7-H3 within the tumor microenvironment may result in the modulation of various components within TME. Such modulation is likely to influence CAR T-cell induced immune interactions, which may enhance or weaken therapeutic immune responses [80]. The use of CAR T-cell therapy to target antigens expressed by medulloblastoma cells is becoming one of the main therapeutic strategies in the search for curative therapy for this fatal disease. Disialoganglioside GD2 is one of the antigens expressed by medulloblastoma with sufficient intensity to allow effective use of CAR T-cell therapy. GD2 is a sialic acid-bearing glycosphingolipid, a member of the ganglioside subfamily, described as a tumor-associated antigen that has emerged as a promising target for aggressive pediatric central nervous system tumors, and its expression on tumor cells and limited expression in normal tissues make it an attractive target for immune therapy. Gangliosides are known to act as regulatory elements in the immune system, in the nervous system, in metabolic regulation, and in cancer progression [81,82]. In a relatively recent study [83], GD2 expression was measured on the primary tumor biopsies of MB children by flow cytometry. According to the authors, such expression was found in over 80% of MB tumors, a high level of expression was observed for the three subgroups, SHH, G3, and G4, while WNT subgroup demonstrated a much lower level of expression. According to this study, CAR.GD2 T-cell therapy in vitro and in vivo models have demonstrated the potential efficacy for the treatment of solid cancers, in particular, for some pediatric CNS tumors that are known to express GD2. Currently, CAR.GD2 T and CAR B7-H3 T constructs are under assessment in a number of ongoing clinical trials for the treatment of medulloblastoma (see Table 2). Figure 2 gives a schematic representation of the various steps leading to decision-making leading to the design of clinical trial to assess cell therapy.

Autologous CAR T-cells therapy has been demonstrated to be a powerful tool for the management of some types of cancer. The potential high therapeutic efficacy of this treatment modality has been demonstrated for hematological malignancies. That said, the extension of highly promising method to other forms of cancer, particularly solid cancers, is hampered by a number of limitations, including high costs, extended manufacturing timelines, and limited accessibility for patients—a limitation which becomes critical for patients with a progressive disease. Other limitations include the availability of target antigen, severe toxic side-effects, and low activity against solid tumors [86,87]. To mitigate some of these limitations, and to enhance the therapeutic potential of conventional CAR T-cells, other CAR-engineered immune cell types are being developed and clinically tested. These include allogeneic CAR T-cells, natural killer (NK), and invariant natural, killer T (NKT) [88,89]. It is interesting to note that the use of invariant natural killer T (iNKT) has been linked to some reduction in GvHD), which may accompany allogeneic hematopoietic stem cell transplantation [90].

#### 1.5.2. Is CAR NK Cell Therapy an Alternative to CAR T-Cell Therapy?

Safety and versatility limitations associated with both autologous and allogeneic CAR T-cells are behind the recent emergence of other variants of cell therapy. In the last few years, chimeric antigen receptor (CAR) NK cell therapy has emerged as an alternative to the well-established CAR T-cell therapy. NK cells have a number of characteristics, which may address a number of limitations associated with CAR T-cells. Unlike T and B cells, which rely on a single somatically rearranged receptor, NK cells use a variety of germline-encoded activating and inhibitory receptors. The access to multiple receptors by these cells enable them to trigger or inhibit functions like cytotoxic granule release and cytokine secretion [91]. Other advantageous characteristics of CAR NK cells include a significantly reduced risk of cytokine release syndrome, and NK cells are not associated with graft-versus–host disease, which render them highly suitable for the future development of scalable allogeneic therapy.

CAR NK therapy is still lagging behind its CAR T counterpart. This observation is highlighted by the number of clinical trials of the first approach (about 120 trials) compared with over 1000 trials using CAR T therapy [92,93,94]. This relatively low number of clinical trials using NK cells renders the therapeutic evaluation of this approach rather immature. That said, data available from these trials give an initial indication of the therapeutic potential of this method. From the careful consideration of the clinical trials in Table 3 and those reported in Ref. [92], the following observations can be made: Most if not all of the reported trials are Phase I studies, involving a relatively low number of patients. As expected, these Phase I studies assessed the safety, tolerability, initial efficacy, and the maximum dose of NK cell tolerated by the patient. Preliminary data generated by some of these trials are encouraging, however, to establish the efficacy of this therapy, we have to await the final conclusions of future Phase II and III clinical trials.

## 2. Conclusions and Future Perspectives

CAR T-cell therapy has demonstrated a notable success in treating various forms of blood cancers. However, this success has not been extended to solid tumors. Such failure is considered one of the main justifications behind the increasing use of natural killer and CAR–macrophage therapies. Based on the investigations discussed in this review, together with various references cited here, a number of observations can be made, and such observations refer to the case of medulloblastoma. (i) It can be said that targeting antigens expressed by medulloblastoma cells using CAR T and NK cell therapies represents the most promising therapeutic strategy in the search for curative, non-aggressive therapy for this devastating disease. However, this objective is faced by two main difficulties: A limited number of antigens, which are expressed with sufficient intensity to allow for the effective use of CAR-based therapy, and a second, more challenging obstacle is the non-permeability of the blood–brain barrier. This obstacle is not limited to medulloblastoma, but extends to all brain cancers. It is needless to point out that, for any therapeutic strategy to have therapeutic success, such a strategy has to ensure that its therapy is reaching its destination. However, artificial intervention to modify permeability of the BBB may increase the danger of serious CNS diseases, such as ischemic stroke, Parkinson’s disease, and Alzheimer’s disease. (ii) Data generated by some ongoing clinical trials suggest that, compared to CAR T-cell therapy, NK cell therapy has lower rates of adverse effects, such as cytokine release syndrome, immune effector cell-associated neurotoxicity syndrome, and graft-versus-host disease. Currently, there are substantial research efforts to enhance the performance of NK cells, including broadening their therapeutic targets, increasing persistence of the injected cells, and the optimization of the production process to facilitate scalability and accessibility of this form of therapy. (iii) Safer and more effective therapies for medulloblastoma patients remains an unmet clinical need. Most ongoing clinical trials investigating NK cell therapy are Phase I studies involving a very limited number of subjects. Currently, there is an urgent need for more clinical trials which enroll a higher number of patients and provide much needed information on various mechanisms of resistance, particularly on the impermeability of BBB. That said, the limited success of cell therapy in treating medulloblastoma cannot be exclusively attributed to the impermeability of BBB, and limited success has been reported for solid tumors in organs not protected by BBB, which means that resistance in medulloblastoma is a multifactorial phenomenon and has to be treated as such.

## Figures and Tables

**Table 1:** Potential drivers of MB resistance to therapy—brief comments and references.

Drivers of MB Resistance to Therapy	Comments/Refs.
Permeability of blood–brain barrier (BBB).	The Impermeability of BBB to a wide range of chemotherapeutic agents is considered a major driver of MB resistance to therapy. As well as the structural permeability of BBB, the expression of efflux transporter proteins on the surface of CNS endothelial cells is another factor which contributes to the prevention of various therapeutic agents from reaching the targeted area within the brain [55,56].
Cellular plasticity	Cellular plasticity is an important driver of therapy resistance. Cancer cells can alter their lineage to evade targeted therapy. To evade therapy, these cancer cells revert to a stem-like state. Such a phenomenon has been described as self-renewal to evade therapy. These studies also observed that, although many genomic aberrations have been associated with lineage plasticity, few tumors exhibit such alterations, suggesting that resistance caused by lineage plasticity occurs through alternative mechanisms [46,57].
Glioblastoma stem cells	Glioblastoma stem cells represent one of the main challenges for attempts to develop efficacious therapies for solid CNS tumors. These tumor initiation cells are characterized by self-renewal, proliferation, and differentiation capabilities. These characteristics render this class of cell a major player in MB resistance and disease recurrence. These stem cells are also characterized by their low abundance within the tumor and low proliferative activity, which protects them from therapies targeting dividing cells [56,58,59].
Anti-apoptotic proteins	Evasion of apoptosis is a hallmark of cancer cells, which is known to contribute to therapeutic resistance in various solid tumors. In the case of MB, a number of proteins have been identified as key players in cell apoptosis and associated pathways. These proteins include BCL-2, MCL-1, and BCL-XL. Bax and Bak are two nuclear-encoded proteins present in higher eukaryotes that are able to pierce the mitochondrial outer membrane to mediate cell death by apoptosis [60,61].
Genetic aberrations	A small subset of subgroups 3 and 4 medulloblastoma patients harbor gene oncogenic drivers, including *MYC* and *MYCN* amplifications, as well as PRDM6 overexpression. On the other hand, the majority of patients within the same subgroups displayed recurrent, large-scale copy number changes. Expression of genes that modulate metabolic responses and energy production (*IDH1*, *HK2*, *HSPH1*, *GLS*, and *NFE2L1*) are increased in recurrent tumors [48,62,63,64].
Altered Molecular pathways	Growth and progression of various forms of cancer, including medulloblastoma, have always been linked to the activation of various pathways. For example, the IL-6/STAT3 pathway has been associated with tumorigenesis and acquired resistance in Group 3 medulloblastoma. Inhibition of STAT3 has been shown to render medulloblastoma cells sensitive to chemotherapy, leading to improved treatment outcomes [65,66].
metabolic reprogramming	Metabolic pathways are used by medulloblastoma cells to adapt to environments which lack the required nutrients and oxygen. Such adaptability is achieved through modulating glucose, lipids, amino acids, and nucleotide metabolism. With regard to MB, such pathways and their impact on resistance to therapy are still understudied. Single-cell multomic analyses showed that in vivo modeling of radiation resistance exhibited chromatin-based metabolic reprogramming focused on wild-type isocitrate dehydrogenase (IDH1) activity. IDH1 inhibition reversed resistance-mediated chromatin changes and resulted in radiation re-sensitization [46,67].
molecular/histological heterogeneity	Recent use of single-cell RNA-seq is providing a much clearer picture on the role of intratumorally heterogeneity and tumor origin for the four molecular subgroups of medulloblastoma. Molecular profiling of MB at the single-cell level emphasized the central role of clonal heterogeneity in resistance to therapy. The impact of heterogeneity on therapy outcome was investigated using quantitative profiling of global proteomes and phospho-proteomes of medulloblastoma samples. These measurements showed that post-translational modifications of *MYC* were associated with poor outcomes in Group 3 tumors [6,56,68].

**Table 2:** Ongoing trials to assess CAR T-cell therapy in targeting certain antigens expressed by medulloblastoma cells.

Trial’s Identifier/Sponsor	Trial Objective	Observations/Refs.
NCT05298995/Gesù Hospital and Research Institute, Italy	Evaluate the safety and therapeutic efficacy of CAR.GD2 therapy in high-risk medulloblastoma patients.	This is a Phase I clinical trial, designed to assess the safety and efficacy of 4.1BB-CD28 CAR T-cell treatment targeting GD2 in pediatric or young adult patients affected by relapsed/refractory malignant central nervous system (CNS) tumors: started 2023 (estimated), primary completion 2027 (estimated), completion 2038 (estimated). Ciccone et al., 2024 [83].
NCT07087002/Stanford University, USA	Evaluate GPC2 Chimeric Antigen Receptor T (GPC2-CAR T) cells for the treatment of relapsed or refractory medulloblastoma in children and young adults	Single-site, open-label Phase 1 clinical trial to evaluate safety and efficacy of autologous GPC2-targeted chimeric antigen receptor CAR-T cells in children and young adults with relapsed or refractory medulloblastoma or other eligible central nervous system (CNS) embryonal tumors. Study started August 2025, primary completion (estimated) August 2027, study completion (estimated) August 2027 [84].
NCT07390539/Robbie MajznerDana-Farber Cancer Institute, USA.	The purpose of this research study is to test the safety and effectiveness of a cell therapy at different doses for children and young adults with recurrent or progressive brain tumors.	This is a single-institution, Phase 1/1b, open-label study, which uses B7-H3 CAR T-cells therapy to treat some forms of recurrent or progressive brain tumor. Study start July 2026 (estimated), primary completion August 2030 (estimated), completion August 2032 (estimated).
NCT0272457/St. Jude Children Hospital, USA	The main objective of this Phase II clinical trial is to assess progression-free survival of a group of WNT medulloblastoma patients treated with different modalities, including a reduced dose of radiation.	This is Phase II study with different primary objectives, including assessment of the progression-free survival distribution of WNT-medulloblastoma patients treated with Stra-tum W1 with reduced-dose craniospinal irradiation and reduced-dose cyclophosphamide. To estimate progression-free survival distribution of Non-WNT Non-SHH medulloblastoma patients treated on Stratum N1 with reduced dose cyclophosphamide. Study start: June 2013(actual). Primary completion (estimated): October 2028. Study completion (estimated) October 2031 [85]

**Table 3:** Some ongoing clinical trials to assess the safety and efficacy of natural killer cell therapy in treating various forms of cancer.

Trial’s Identifier/Sponsor	Observations
NCT05020678/Nkarta, Inc.	This is a single arm, open-label, multi-center, Phase I study to determine the safety and tolerability of an experimental therapy called NKX019 (allogeneic CAR NK cells targeting CD19) in patients with relapsed/refractory non-Hodgkin lymphoma, chronic lymphocytic leukemia, or B cell acute lymphoblastic leukemia. Study Start: August 2021, primary completion, March 2025 (estimated), study completion December 2038 (estimated), enrollment 150 (estimated).
NCT05194709/Wuxi People’s Hospital	This study is an interventional, single arm, open-label to evaluate the safety, tolerability, initial efficacy, and pharmacokinetics of anti-5T4 CAR-NK cells in patients with advanced solid tumors. Study start: December 2021, primary completion, December 2022 (estimated), study completion December 2022, enrollment 40 (estimated).
NCT02271711/M.D. Anderson Cancer Center	This is a Phase I study to assess the safety, efficacy, side-effects and maximum tolerated dose of administering autologous natural killer (NK) cells. These cells were administered directly into the ventricle in recurrent/refractory malignant posterior fossa tumors. Study start: March 2015, primary completion August 2020, study completion August 2020, enrollment 12.
NCT05588453/Kari Kendra, (Ohio State University Comprehensive Cancer Center)	This Phase I/II trial is to assess the safety and tolerability of UD TGFbetai NK cells in combination with temozolomide as a lymphodepleting agent in patients with metastatic melanoma to the brain and to determine the recommended Phase II dose (RP2D) (Phase I). Phase II: to determine the intracranial response rate (Phase II). Study start: March 2023, primary completion, April 2026, study completion April 2026 (estimated), enrollment, 30 (estimated).
NCT05962450/Beijing YouAn Hospital	The main objective of this Phase II clinical trial is to assess the efficacy and safety of autologous iNKT cells in patients with progressed hepatocellular carcinoma (HCC) after treatment with PD-1 antibody. Study starts October 2023 (estimated), primary completion August 2025 (estimated), study completion August 2025 (estimated), enrollment (estimated) 84.

## Data Availability

No new data were created or analyzed in this study. Data sharing is not applicable to this article.

## Abbreviations

ICIs	Immune checkpoint inhibitors
MB	Medulloblastoma
PTMs	post-translational modifications
CAR T	Chimeric antigen receptor T cell therapy
CAR NK	Chimeric antigen receptor natural killer cell therapy
CTCs	Circulating tumor cells
ctDNA	Circulating tumor DNA
PCR	Polymerase chain reaction
NGS	Next-generation sequencing
CSF	Cerebrospinal fluid
MS	Mass spectrometry
LC	Liquid chromatography
LC-MS/MS	Liquid chromatography/tandem mass spectrometry
scFv	Single-chain variable-fragment
PPP	Pentose phosphate pathway
TKT	Transketolase
ELISA	Enzyme-linked immunosorbent assay
BBB	Blood–brain barrier
FDA	Food and Drug Administration
EMA	European Medicines Agency
PD-L1	Cell death ligand-L1
PD-1	Programmed cell death-1
CTLA-4	Cytotoxic T lymphocyte antigen-4
LAG-3	Lymphocyte activation gene-3
B7-H3	B7 homolog 3 protein
BTLA	B and T lymphocyte attenuator
TIGIT	Cell immunoglobulin and ITIM domain
ICOS	Inducible T cell costimulatory
VISTA	V-domain immunoglobulin suppressor of T cell activation
GvHD	Graft-versus-host disease
TME	Tumor microenvironment
